# Acupuncture for primary fibromyalgia: Study protocol of a randomized controlled trial

**DOI:** 10.1186/s13063-020-04317-y

**Published:** 2020-06-17

**Authors:** Man Han, Jiakang Cui, Yuya Xiao, Donghong Xiao, Juan Jiao, Qiuwei Peng, Feng Tian, Xiaopo Tang, Jianyong Zhang, Quan Jiang

**Affiliations:** 1 Department of Rheumatology, Guang’anmen Hospital, China Academy of Chinese Medical Sciences, Beijing, China; 2 Department of Rheumatology, Shenzhen Traditional Chinese Medicine Hospital, Shenzhen, China; 3 Beijing University of Chinese Medicine, Beijing, China; 4 Beijing CreateMed Medicine Technology Co., LTD, Beijing, China

**Keywords:** Acupuncture, Primary fibromyalgia, Randomized controlled trial, Pain, Fatigue

## Abstract

**Background:**

Acupuncture is well recognized for its unique therapeutic effect for many diseases as a nonpharmacological therapy in traditional Chinese medicine (TCM). However, whether acupuncture can effectively treat fibromyalgia is currently unclear. Therefore, we aim to design a study protocol of a randomized controlled clinical trial and assess the effectiveness of acupuncture for patients with fibromyalgia, which may lead to alleviation of clinical symptoms and improvement of patients’ quality of life.

**Methods:**

The study is designed as a randomized, blinded, placebo-controlled trial of two cohorts conducted at Guang’anmen Hospital of China Academy of Chinese Medical Sciences and Shenzhen Traditional Chinese Medicine Hospital, respectively. A total of 68 patients with primary fibromyalgia, diagnosed with the American College of Rheumatology criteria, are randomly allocated with a 1:1 ratio to acupuncture or sham acupuncture groups. All subjects will receive acupuncture intervention for 8 weeks with follow-up assessments every 4 weeks for 16 weeks. The primary outcome will be evaluated using the visual analogue scale (VAS) and revised fibromyalgia impact questionnaire (FIQR) for pain intensity. The secondary outcome measures will include: Multidimensional Assessment of Fatigue scale (MAF), Short Form-36 (SF-36), Beck Depression Inventory (BDI), Pittsburgh Sleep Quality Index (PSQI), Chinese perceived stress scales (pss-14), changes in the number of 18 tender points, patient satisfaction for the treatment and adverse events. The mentioned outcome measurements will be assessed every 4 weeks for 6 months.

**Discussion:**

This clinical trial will use advanced research methods to evaluate the efficacy and safety of acupuncture on fibromyalgia. The results of this trial may provide clinical evidence on the beneficial effects of acupuncture in treating fibromyalgia.

**Trial registration:**

Chinese Clinical Trial Registry, ChiCTR1800016826: AMCTR-IOR-18000184. Registered 27 June 2018, http://www.acmctr.org/listbycreater.aspx

## Background

Primary fibromyalgia (FM) is a chronic clinical condition characterized by diffuse pain and stiffness as the major clinical feature, which is often accompanied by fatigue, weakness, sleep disorders, emotional abnormalities, and cognitive dysfunction [1]. The prevalence of FM is about 2–8% with the male-to-female ratio of about 1:9 [2, 3]. The clinical manifestations of FM are complex and diverse, including persistent pain, fatigue, and sleep disorders [4].

Although the etiology of FM remains unclear, it is currently considered that both neuroendocrine changes and immune dysregulations are involved in the pathogenesis of this disease [3]. Current treatments of FM mainly include pharmacotherapy and nonpharmacologic therapy. Antidepressants are the choice of medication for the treatment of FM, among which the tricyclic antidepressant amitriptyline is the most widely used. The second-generation anticonvulsant pregabalin is the first drug approved by the FDA for the treatment of FM. Other therapeutics including reuptake inhibitors, highly selective monoamine oxidase inhibitors, and analgesics. Non-opioid central analgesic tramadol has also shown certain effects in treating FM [5]. However, the overall therapeutic effects of the above drugs are considered to be less than satisfactory since only a small portion of patients could receive the expected benefits [6]. Currently, the available treatments in traditional Chinese medicine (TCM) comprise diverse and effective nonpharmacologic therapies such as acupuncture, massage, cupping, tai chi, and qi gong. Notably, the newly revised FM treatment recommendations by EULAR have placed acupuncture as one of the specific recommendations for the nonpharmacologic therapies since acupuncture has been shown to alleviate pain, relieve fatigue, and improve the quality of life in patients with FM [7].

Acupuncture has been used in medical practice with little side effects and low cost for more than 2000 years in China and is now widely recognized around the world. Based on the meridian theory, acupuncture therapy could promote flow of qi and blood circulation and regulate the balance of Yin and Yang, which has been used in the treatment of many diseases and clinical syndromes. A large number of studies have demonstrated the efficacy and advantages of acupuncture therapy in alleviating chronic pain [8], insomnia [9], anxiety, and depression [10]. Acupuncture exhibits a rapid pain-relieving effect for the chronic soft tissue diseases, which has been confirmed by research findings on its anti-inflammatory and analgesic effects and improvement of microcirculation [11, 12]. Although acupuncture has shown certain efficacy and safety in treating FM cases, several studies have also reported no significant difference between acupuncture therapy and placebo treatment [13–15].

Thus, this study aims to provide a protocol for clinical trials with randomized controlled studies to confirm the therapeutic effects of acupuncture in the treatment of FM.

## Methods

### Study design

This study is a two-center, blinding, randomized, controlled trial with two arms for 8 weeks. A total number of 68 patients with FM who meet the criteria are randomly divided into acupuncture group and sham acupuncture group according to the ratio of 1:1. The intervention will be performed for 8 weeks with a follow-up of 16 weeks. The treatment of acupuncture and placebo is three times a week for the first 4 weeks and twice a week for the next 4 weeks. This clinical trial will be completed at Guang’anmen Hospital of China Academy of Chinese Medical Sciences and Shenzhen Traditional Chinese Medicine Hospital. This study will follow the relevant regulations of the World Medical Congress “Helsinki Declaration” and has been approved by the Ethics Committee of Guang’anmen Hospital and Shenzhen Traditional Chinese Medicine Hospital. All enrolled subjects will be informed with a complete and comprehensive introduction including the purpose, procedure, and possible risks of the study. The informed consent will be obtained before the start of this study.

### Participants

#### Inclusion criteria

Participants who meet the following criteria will be included:

- ▪ Meet the American College of Rheumatology criteria for FM in 1990 [16];
- ▪ Age not less than 18 years old;
- ▪ No acupuncture contraindications;
- ▪ Informed consent obtained;
- ▪ No FM medication or stop FM medication for at least 2 weeks.

#### Exclusion criteria

Subjects who fulfill any of the following criteria will be excluded:

- ▪ Subjects with severe systemic disorders (lymphoma; central nervous system, renal, or pulmonary involvement; myositis or vasculitis), renal or liver failure;
- ▪ Subjects with systemic or local acute infection;
- ▪ Subjects with complications of other rheumatic diseases;
- ▪ Subjects with the pain visual analog scale score less than 4.0 cm/10 cm;
- ▪ Pregnant women;
- ▪ Currently lactating women;
- ▪ Women planning to become pregnant during the study period;
- ▪ Subjects currently participating or having participated in another trial during the last 30 days prior to the initial screening examination;
- ▪ Subjects having received acupuncture treatment.

### Participant recruitment

This randomized, sham-controlled, patient and assessor blinded trial will be conducted in Guang’anmen Hospital and Shenzhen Traditional Chinese Medicine Hospital, which will provide outpatient service for FM. Participants in this study will be recruited from either outpatient clinic or inpatient department via advertisement at hospital-based electronic social media platform and poster distribution in the public areas of hospitals and nearby communities with the details of this study and contact information.

The interested subjects will be advised to contact the research assistants by phone or on site for initial screening. The potential participant is registered and given an appointment with the assigned physician for checking the inclusion and exclusion criteria. Patients who meet all inclusion criteria but no exclusion criteria will be invited to participate in the study and provided with the details of this RCT. All the participants will be asked to sign an informed consent form with their comprehensive information collected. The participants will be randomly assigned into two groups: the acupuncture group and the sham acupuncture group. All patients will not be informed about which group they have been assigned to in order to maintain blinding in this trial. Then, the baseline assessments will be performed by the same physician and recorded in CRF. All participants will undergo 8-week treatment and 16-week follow-up with relevant assessments every month in CRF. Each participant will be given a schedule for the dates of intervention and follow-up appointments performed by a research assistant who is neither involved in the treatment nor in the randomization.

### Randomization and blinding

The study is a two-center, double-blind, and placebo-controlled trial. With the block randomization method, the random number table will be generated by an independent third party, the Hospital’s Clinical Evaluation Center, using SAS 9.4 software (SAS Institute, Cary, NC, USA). The randomized assignment sequence is placed in a sealed opaque envelope whereas the blind codes are kept at the department of research management and can be reproduced when needed. All of the patients participated in this study; principal investigators and research staff will remain blinded to all patients’ randomization assignments throughout the duration of this study. Eligible patients will be randomly allocated to either the acupuncture or sham group according to a 1:1 ratio.

### Intervention

All participants will receive either real acupuncture or sham acupuncture for 8 weeks. The frequency of acupuncture intervention is three times per week for the first 4 weeks and twice a week for the following 4 weeks. Every participant will be placed in a separate room to receive the treatment for 30 mins every time. All acupuncturists participating in the study are licensed medical practitioners to ensure the accuracy and consistency of the treatments provided. Meanwhile, adverse reactions that may occur during the study will be recorded.

### Acupoints and needles

**Fig. 1:**
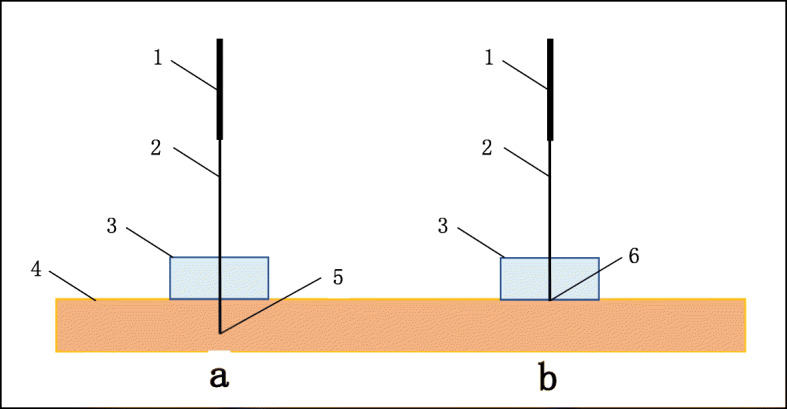
The comparison of acupuncture treatment and sham acupuncture. **a** real acupuncture. **b** sham acupuncture.1. Needle handle. 2. Needle body. 3. Adhesive pad. 4. Skin. 5.Sharp tip. 6. Blunt tip. Needles: Both real and sham needles (Shanghai Zhenkang Medical Technology Company, Ltd., Shanghai, China) are stainless-steel needles, which have the same size of 0.30 × 40 mm according to the depth of acupoints’ site in this trial

Acupoints include three on the head, four on the back, two on the upper extremities, and eight on the lower extremities. Before acupuncture treatment, a small pad will be pasted on each acupoint. All the needles will be inserted in the acupoints through these pads (Fig. 1).

### Description of sham acupuncture

The sham needle consists of a needle with a blunt tip and a circle adhesive pad (Fig. 1). The needle is very similar to the real needle, 0.30 mm wide, and 40 mm long. The adhesive pad is made of the sterile cylindrical polyethylene foam (10 mm in diameter and 5 mm in length) with a double-sided adhesive tape at the bottom. The adhesive pad supports the sham needle on the acupoint and ensures the implementation of blinding during the intervention. Thus, the sham needle with a blunt tip only contacts the skin surface but will not penetrate into the skin of the participants during sham acupuncture.

### Process of the treatment

**Fig. 2:**
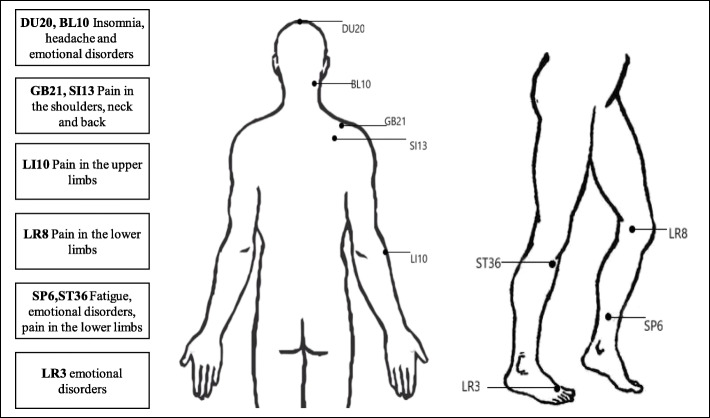
The accupoints adopted in the study and their indications

Each participant will be interviewed by an investigator prior to the commence of treatment and fill out the CRF with a series of initial data at baseline of the study. After skin sterilization, an adhesive pad made of aseptic cylindrical polyethylene foam with double-sided tape on the bottom is placed on the acupuncture points for fixing the needle and implementation of blinding. The points and manipulation of acupuncture in both groups are the same, with Baihui (DU20), bilateral Tianzhu (BL10), bilateral Jianjing (GB21), bilateral Quyuan (SI13), bilateral Shousanli (LI10), Ququan (LR8), and bilateral Taichong (LR3) with even method, bilateral Sanyinjiao (SP6), Zusanli (ST36) with reinforcing method (Fig. 2). Needles will be retained for 30 min during treatment. Following needle insertion, the state of “de qi” (feelings of patients including soreness, numbness, heaviness, or tingle at the points) is achieved by twirling, lifting, and thrusting, which is essential for the effective treatment [17].

### Combination therapy

#### Drugs allowed to be combined

- ▪ Chinese medicine including traditional Chinese medicine decoction and Chinese patent medicine) used for at least 2 weeks with stable dosage and type;
- ▪ Drugs for cardiovascular and cerebrovascular diseases and the like used for at least 6 months with stable dosage and type;
- ▪ All the combined medications are required to be recorded with detailed information.

#### Drugs not allowed to be combined

- ▪ Non-steroidal anti-inflammatory drugs;
- ▪ Oral corticosteroids;
- ▪ Opioid analgesics.

### Outcome measurements

#### Primary outcomes

1. The visual analogue scale (VAS) [18] for pain intensity: Using a horizontal ruler of 100 mm, “0” representing no pain and “100” representing unbearable pain. The pain VAS intensity will be measured at the baseline, week 4, week 8. A total of seven assessments will be measured monthly during the 8-week treatment period and the follow-up period at week 12, 16, 20, and 24.
2. Revised Fibromyalgia Impact Questionnaire (FIQR) [19] for pain intensity: FIQR assesses the health status of patients with fibromyalgia, including physical function, working condition, depression, anxiety, pain, stiffness, fatigue, sensitivity, and well-being. The scoring system ranges from 0 to 100, in which a higher score represents a heavier state of FM. Each subject will be asked to complete the questionnaire before treatment based on the overall situation of the past 24 h [20].

#### Secondary outcomes

1. Multidimensional Assessment of Fatigue scale (MAF): The MAF scale covers 16 items that measure five dimensions of fatigue: degree, severity, distress, degree of interference with activities of daily living, and timing. Items 1–14 contain ten-point numerical rating scales and items 15–16 have multiple-choice responses. The ten-point numerical rating scale ranges from 1 to 10 [21].
2. The MOS 36-item short-form health survey (SF-36): It is a comprehensive scale reflecting the quality of life containing contents of eight domains: physical functioning, role-physical, bodily pain, general health, vitality, social functioning, role-emotional, and mental health. Moreover, it has an independent item reporting health transition during the past 1 week [22].
3. Beck Depression Inventory (BDI) [23]: The scale was used to assess the severity of depressive symptoms. It contains 21 entries, each of which is scored from 0 to 3. The scale is divided into a total score of 21 items. The severity of depression is divided into four degrees by the total score. The total score between 0 to 13 represents no depression, 14 to 19 mild depression, 20 to 28 moderate depression, and 29 to 63 severe depression.
4. Pittsburgh Sleep Quality Index (PSQI) [24]: It consists of subjective sleep quality, sleep time, sleep efficiency, sleep disturbance, whether or not to use hypnotic drugs and effects on daytime function. It has a total of seven items, with score 0–3 for each. The higher the cumulative score, the worse the quality of sleep.
5. Chinese perceived stress scale (pss-14): This scale is to assess the patient’s personal feelings and thoughts in the past month. It consists of 14 items that each item has six response options ranging from “All of the time” to “None of the time” [25].
6. Changes in the number of 18 tender points: The pressure applied to each tender point was 4 kg/cm^2^ [26]. The patients will be asked to inform the investigators when tenderness occurred while the total number will be calculated and compared to the baseline.
7. Patient satisfaction for the treatment: Patient satisfaction will be assessed by Patient Global Impression of Change (PGIC) [27] after 8 weeks of treatment, which is a PRO counterpart to the clinical assessment by doctors. It consists of one item taken from the Clinical Global Impressions scale (CGI) and adapted to the patient. The levels of satisfaction will be measured with seven scales ranked from 1 to 7, 1 is for “Very much improved” and 7 for “Very much worse”.

Adverse events will be monitored during the 8 weeks of treatment. Any adverse reactions occurred during the study will be documented in the “Adverse Reaction Form” while the patients are followed up until the symptoms disappear or the abnormal indicators return to normal. In the event of a serious adverse event, the necessary measures will be taken immediately for the safety of the subject.

**Fig. 3:**
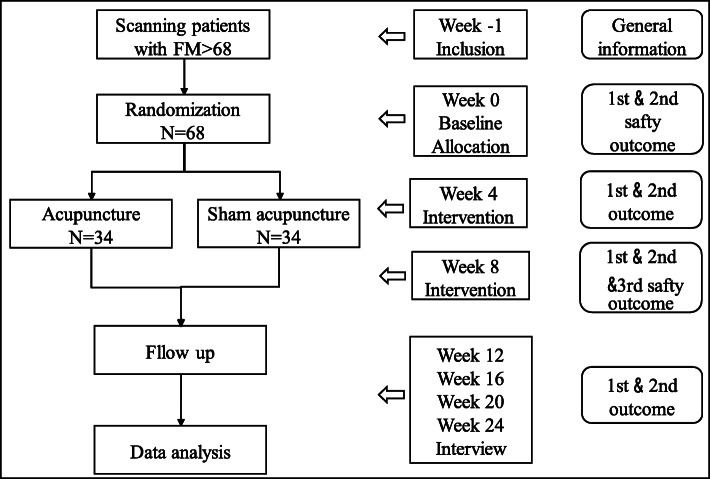
Trial flow and study design. General information = name, gender, age, height, weight, duration of illness, smoking history, drinking history, education, job, family income. 1st outcome = pain-VAS, Revised Fibromyalgia Impact Questionnaire (FIQR). 2nd outcome = Multidimensional Assessment of Fatigue scale (MAF), SF-36, pss-14, Beck Depression Inventory (BDI), Pittsburgh Sleep Quality Index (PSQI), changes in the number of 18 tender points. 3rd outcome = Patient satisfaction for the treatment (PGIC)

**Fig. 4:**
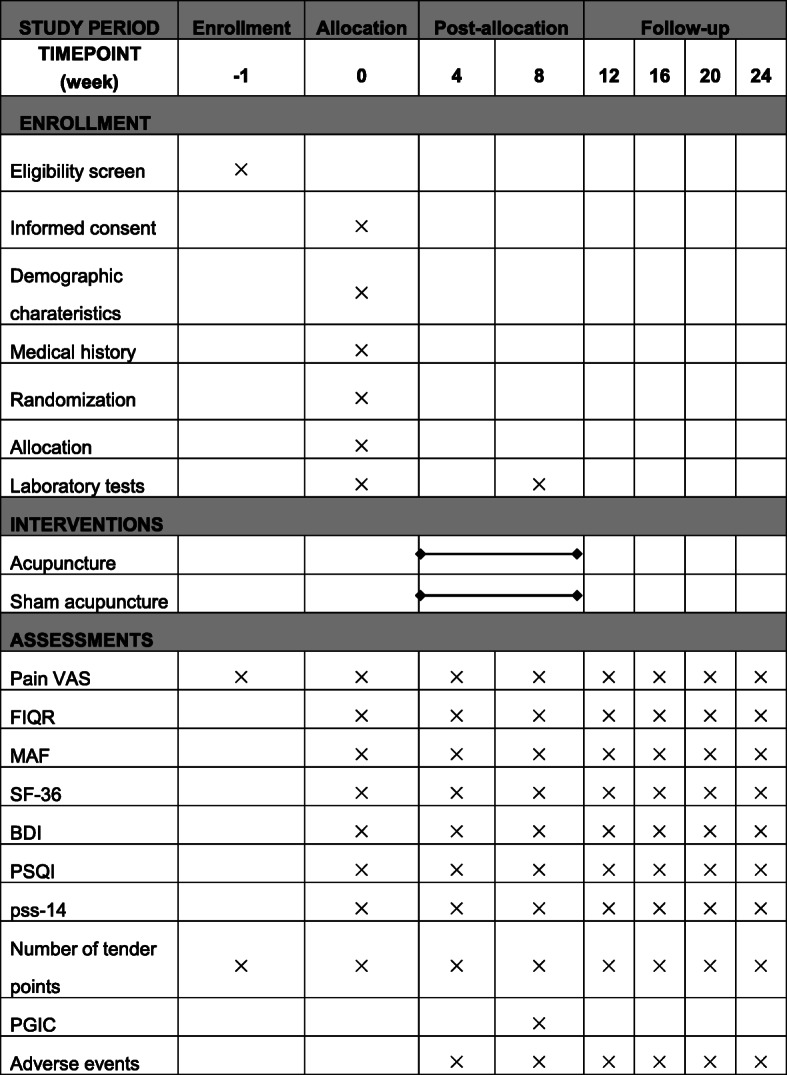
SPIRIT figure of enrolment, interventions, and assessments. *FIQR* Revised Fibromyalgia Impact Questionnaire, *MAF* Multidimensional Assessment of Fatigue scale, *SF-36* The MOS 36-item short-form health survey, *pss-14* Chinese perceived stress scale, *BDI* Beck Depression Inventory, *PSQI* Pittsburgh Sleep Quality Index, *PGIC* Patient satisfaction for the treatment

Primary and secondary efficacy indicators will be evaluated every 4 weeks except the PGIC assessed after 8 weeks of treatment. Safety evaluations will be conducted at baseline and week 8 including blood pressure, breathing, heart rate, blood routine, urine routine, liver, and kidney function (ALT, AST, Cr, BUN), Electrocardiogram (Figs. 3 and 4).

### Sample size

The calculation for sample size was based on the changes in pain VAS scores. We adopted the method described in previous studies [28, 29] that the changes are − 4.00 in the acupuncture group and − 2.50 in the sham-acupuncture group, respectively, indicating that the mean difference between two group is 1.5 with standard deviations of 2.55 and 1.25. Given that the ratio of the acupuncture group and the control group is 1:1, the required sample size is 29 cases for each group as estimated by SAS 9.4 software using a two-sided test with a significance level (α) of 0.05 and a power (1-β) of 0.80. With a possible dropout rate of around 15%, the acupuncture group and control group require a total of 68 cases.

### Quality control

The standard operation procedure (SOP) for the acupuncture operation method is established. The identification, registration, and treatment of participants will be managed by the SOP. Training of acupuncture theory and surgical aseptic operation for acupuncturist with certain clinical experience will be conducted to ensure the consistency of repeated operations of acupuncture. A corresponding data security monitoring plan will be developed. All adverse events are recorded in detail, properly processed, and tracked until properly resolved or the condition is stable, and reported to the Ethics Committee in a timely manner.

### Trial monitoring

The Trial Steering Committee is established for monitoring the trial conduct and ensuring the safety and quality of the data, which consists of three members including a senior rheumatologist, an acupuncturist, and a statistician. The committee is independent from the study group and has no conflict of interest with the investigators. They will provide regular supervision and hold monthly meetings (either face to face or online) to ensure that the trial is conducted smoothly and ethically. The committee will also be responsible for monitoring data collection to ensure the authenticity and integrity of the data. They will conduct at least one on-site visit every 6 months during the process of the trial. During the visit, they will interview investigators, examine the original research documents, check the participant enrollment, and confirm the compliance of clinical centers with the trial protocol. Moreover, they will identify the problems in the trial and provide recommendations on the amendment of the protocol. If any decisions on changes of the protocol were to be made, they would notify the funder first. Subsequently, the PI will apply for approval from the Ethics Review Board with written application and notify the investigators with written notice upon receiving the approval from the ethics committee. Any deviations from the protocol will be fully documented using a breach report form. In addition, the protocol will be updated immediately in the clinical trial registry.

### Data management

The data of this clinical trial is managed by Beijing CreateMed Medicine Technology Co., LTD, which ensures the authenticity, integrity, and privacy of clinical test data during the research process. The clinical trial database is structured with EpiData 3.1 (Chinese version) by the appointed data manager who is responsible for the regular database management and maintenance. All of the data from the research centers will be imported into the clinical trial database by two research assistants. Any missing or incorrect data will be detected by software system. In such case, the original CRFS will be checked to correct or complete every piece of data.

### Statistical analysis

For statistical analysis, mean ± standard deviation (M ± SD) is used for continuous variables which meet normal distribution. Chi-square or Fisher’s exact test will be performed to compare baseline characteristics for categorical variables. The primary outcome variable between the two groups will be analyzed by Chi-square test or Fisher’s exact test, while the analysis of independent-samples *t* test or Wilcoxon rank-sum test will be used for secondary outcomes.

To assess the effect of acupuncture therapy for fibromyalgia, intention-to-treat (ITT) [30] analysis will be performed for statistical analysis. A value of *P* < 0.05 with two-tailed test will be considered statistically significant.

### Publication policy

The results will be released by the study group after the data analysis. All presentations and publications are expected to protect the integrity of the major objectives of the study. Any data that break the blind will not be presented prior to the release of mainline results. Recommendations as to the timing of presentation of such endpoint data and the meetings at which they might be presented will be discussed by the Steering Committee. The primary outcomes will be presented via publications including papers, abstracts in the meetings, and announcement in the clinical trial registry. The datasets analyzed during the current study are available from the corresponding author upon request.

### Ethical considerations

The protocol has been approved by the Institutional Ethics Committee of Guang’anmen Hospital (approval number: 2018–060-KY) and Shenzhen Chinese Medicine Hospital (approval number: Shenzhen TCM Hospital Ethical Review for Scientific Research-2018-54). All subjects will be required to sign the informed consent form. The consent form is available from the corresponding author upon request. In the consent form, participants will be asked if they agree to use of their data should they choose to withdraw from the trial. Participants will also be asked for permission for the research team to share relevant data with members from the universities involved in the research. This trial does not involve collecting biological specimens for storage. Personal information of the enrolled participants will be maintained properly in order to protect confidentiality before, during, and after the trial.

## Discussion

For FM, extensive pain and physical discomforts are the main clinical manifestations, which severely affect the quality of life for patients. It is estimated that about 90% of patients have received supplemental replacement therapy [31]. Acupuncture is a unique nonpharmacologic therapy. Since there are contradictory findings about the effectiveness of acupuncture therapy for the treatment of FM [32, 33], here we have designed a randomized controlled trial protocol to assess the efficacy of acupuncture in the treatment of FM mainly by assessing the alleviation of clinical symptoms.

In TCM, FM is named as “Jin bi” that belongs to the “Bi syndrome”, which is also the synonym of rheumatism. According to the theory of TCM, the disorder of Qi movement and stagnation of Qi and blood contribute to the development of this disease. The acupoint selection is designed based on syndrome differentiation combined with the theory of meridians under the holism. In this study, we mainly chose the acupoints that regulate the stagnation of Qi and blood and also select the acupoints corresponding to the tender points along the meridians, which include Tianzhu, Jianjing, Quyuan, ShouSanli, and Ququan. In line with the principle of dialectical relation between regional part and whole body, other selected acupoints include Baihui, Sanyinjiao, Zusanli, and Taichong.

As a nonpharmacologic therapy, acupuncture may exert its effects on FM by local stimulation to regulate systemic function with a low safety risk to patients. However, there are some reports on the possible side effects of acupuncture. The common traumatic complications include pneumothorax, but most complications could recover spontaneously or after treatment. Occasionally, acupuncture may cause infection. Moreover, it has been reported that acupuncture may trigger secondary cardiac tamponade [34]. Therefore, all participated acupuncturists need standard training before the commerce of the study and will operate strictly according to the SOP in order to ensure subjects’ safety during the treatment.

We choose a type of blunt-pointed sham needle with a similar shape to the actual needle in this study. During treatment, the sham needles will penetrate through the adhesive pad reaching the skin surface but will not be inserted into the tissue so that the subjects could still feel the pain of “acupuncture”, which ensures the treatment to be conducted in a blinded manner. In a randomized controlled previous study, 60 subjects were randomized to acupuncture group and placebo group in which this kind of sham needles were used [35]. The results showed that there was no significant difference in acupuncture perception between the placebo group and the acupuncture group [33]. For this study, we will take further measures to minimize the technical discrepancies. For example, patients who have received acupuncture treatment will not be included because they may notice the subtle difference in real and sham acupuncture based on their experience. In addition, the acupuncturists will not participate in the interviews, whereas the clinical data will be collected in a separate room by other investigators. The data management and statistical analysis will be conducted by the statisticians from the third party.

In this study, there are certain limitations including the limited sample size and research centers participated in the trial. To achieve the successful completion of this clinical trial, the enrolled participants will be local residents from Beijing and Shenzhen, which will ensure their participation during the entire period of treatment. Future studies will aim to increase the sample size and include more research centers to further validate the therapeutic effects of acupuncture on patients with FM.

### Trial status

This protocol version No. A001 revised on Mar 1, 2018 has been approved by the Institutional Ethics Committee of Guang’anmen Hospital and Shenzhen Chinese Medicine Hospital. The date of recruitment began in July 2018 and will be completed in August 2020. This study will be finished in December 2020.

## Data Availability

The Clinical Evaluation Center of Guang’anmen Hospital is responsible for data and safety monitoring, which is also in charge of checking the quality of data collection and statistical analysis. The members of the Clinical Evaluation Center have no conflict of interests in this study.

## Abbreviations

FM: Fibromyalgia
TCM: Traditional Chinese medicine
VAS: Visual Analogue Scale
RCT: Randomized controlled trial
FIQR: Revised Fibromyalgia Impact Questionnaire
MAF: Multidimensional Assessment of Fatigue scale
SF-36: The MOS 36-item short-form health survey
BDI: Beck Depression Inventory
PSQI: Pittsburgh Sleep Quality Index
pss-14: Chinese perceived stress scale
PGIC: Patient Global Impression of Change
CRF: Case Report Form
SOP: Standard operation procedure
ITT: Intend to treat
